# A dual-process psychological model of impulse buying in digital commerce: evidence from livestream and marketplace contexts

**DOI:** 10.3389/fpsyg.2026.1774474

**Published:** 2026-03-24

**Authors:** Qiu Xilai, Tayyeba Bashir, Ammara Naseem, Rana Faizan Gul, Burhan Sadiq

**Affiliations:** 1 Business School of Hohai University, Hohai University, Nanjing, China; 2 College of Economics and Management, Beijing University of Technology, Beijing, China; 3 School of Management, Beijing Institute of Technology, Beijing, China

**Keywords:** algorithmic governance, decision confidence, hedonic motivation, impulse buying, livestream commerce, online marketplaces, recommender systems

## Abstract

The rapid rise of livestream social commerce has reshaped online shopping by combining algorithmic personalization, entertainment, and real-time persuasion. However, how algorithmic quality, algorithmic governance, and hedonic experience jointly influence impulse-buying intentions across different digital shopping contexts remains insufficiently understood. Drawing on dual-process perspectives from persuasion and stimulus-organism-response theories, this study develops a dual-route framework explaining how algorithmic and psychological factors drive impulse buying through cue-driven and confidence-based mechanisms. Survey data were collected from 564 Chinese consumers, evenly split between livestream commerce and conventional online marketplaces. The results suggest selective contextual differences. In livestream settings, algorithmic quality and governance function as salient cues that directly stimulate impulse-buying intentions. In contrast, in marketplace environments, hedonic motivation and algorithmic governance primarily influence impulse buying indirectly by strengthening consumers’ decision confidence. Configurational analysis further identifies multiple sufficient combinations of technological and motivational conditions that lead to high impulse-buying intentions, demonstrating equifinality across contexts. By integrating algorithmic quality and governance into a dual-route impulse-buying model, this study advances understanding of how transparency and system design actively shape spontaneous consumer behavior. The findings also offer practical guidance for designing transparent, confidence-enhancing, and ethically responsible recommendation systems in digital commerce.

## 1 Introduction

With the rapid rise of livestream social commerce in China, consumers increasingly encounter two distinct online shopping environments: immersive, real-time, socially interactive livestream platforms and more traditional catalog-based online marketplaces (Ngo et al., 2025). Although both environments rely heavily on algorithmic decision support, their interface dynamics and psychological demands differ substantially. In livestream sessions, hosts guide purchases in real time amid interactive chat, social presence, and urgency cues. In contrast, in marketplace settings, consumers browse product pages, reviews, and comparison tools at their own pace. Understanding how these contrasting environments shape consumer decision-making is important not only for advancing theory but also for informing platform designs that promote engagement without undermining consumer welfare.

A growing body of research demonstrates that algorithmic quality (AQ) influences user trust and purchase intentions (Lee et al., 2025). Complementing this stream, studies show that hedonic motivation plays a key role in unplanned purchasing in e-commerce contexts (Gallin and Portes, 2024) and more broadly stimulates exploratory and enjoyment-driven consumption (Abbasi et al., 2025). In parallel, decision confidence (DC) has emerged as a proximal psychological driver of purchasing behavior, as believing in the correctness of one’s choice reduces hesitation (Abbasi et al., 2025) and facilitates immediate action. Together, these findings suggest that both system-related cues and internal psychological states are central to impulse-buying behavior in digital commerce.

Alongside quality and motivation, algorithmic governance (AG), including transparency and minimized privacy intrusion, has typically been conceptualized as a trust-enhancing safeguard. However, most existing studies (Alhelaly et al., 2025; Montecchi et al., 2024; Sansome et al., 2024) treat AG primarily as a hygiene factor that prevents user backlash rather than as an active driver of consumer behavior. As a result, prior work rarely examines whether AG can directly stimulate impulse-buying intentions (IBI) or whether its effects operate indirectly through DC. More critically, much of the literature pools across shopping contexts, implicitly assuming that decision mechanisms are invariant across platforms. This assumption overlooks the possibility that consumers process cues differently under the time pressure and social immersion of livestream shopping compared with the more deliberative environment of online marketplaces. Moreover, conventional linear modeling approaches capture average net effects but obscure alternative configurational pathways that may also lead to high impulse buying.

These limitations point to several unresolved research gaps: the absence of context-sensitive mechanistic models that distinguish impulse-buying routes across livestream and marketplace environments, an underexplored role of AG as an active behavioral catalyst, and limited use of configurational approaches to complement linear models in this domain. To address these gaps, this study poses the following research questions:

*RQ1*: Do impulse-buying intentions in AI-mediated commerce emerge through a cue-driven route in livestream settings and through a confidence-based route in conventional marketplaces?

*RQ2*: Does algorithmic governance function as a behavioral catalyst, exerting a direct effect on impulse buying in livestreams and a primarily confidence-mediated effect in marketplaces?

In this study, livestream commerce refers to real-time, host-led selling sessions that integrate product demonstrations, interactive chat, social presence, and urgency cues within an integrated purchase interface (Taobao Live). In contrast, conventional marketplaces are catalog-based platforms in which consumers browse static product listings, reviews, and recommendation modules at their own pace^1^. These environments differ markedly in temporal dynamics and cognitive load. Livestreams intensify social immersion and time pressure, encouraging affective and heuristic processing, whereas marketplaces support deliberation and comparative evaluation, fostering confidence-based reasoning. This contextual distinction provides the foundation for our dual-route theorization of impulse buying.

This study advances impulse-buying research in several ways. First, it proposes a dual-route mechanism showing that impulsive behavior in AI-mediated commerce can arise from either cue-driven heuristics or confidence-based reasoning, depending on platform context. Second, it reconceptualizes AG as a behavioral catalyst that enhances impulsivity by providing psychological safety and legitimacy, extending prior work that framed governance primarily as a restraining or trust-building factor. Third, it identifies DC as a transferable psychological mechanism linking AQ, AG, and hedonic experience to impulsive outcomes, thereby integrating affective, cognitive, and technological antecedents within a unified framework. In addition, by combining Partial Least Squares Structural Equation Modeling (PLS-SEM) and fuzzy-set Qualitative Comparative Analysis (fsQCA), the study uncovers actionable configurations that can inform both managerial design decisions and regulatory assessments of transparent and ethical shopping environments.

Drawing on dual-process frameworks such as the Elaboration Likelihood Model (ELM) (Petty, 1986) and the stimulus-organism-response (SOR) paradigm (Wang et al., 2024), we conceptualize two distinct decision routes to impulse buying. In livestream commerce, consumers are more likely to rely on heuristic, cue-driven processing triggered by vividness, immediacy, and algorithmic signals, analogous to the peripheral route in the ELM. Although grounded in the stimulus-organism-response (SOR) paradigm, the present framework advances impulse-buying research in three important ways. First, rather than treating internal states as a unified affective construct, we distinguish between affective immediacy and cognitive assurance as qualitatively distinct organism-level mechanisms rooted in dual-process theory. Second, we identify algorithmic governance as a structural feature of digital platforms that activates reflective processing, thereby positioning algorithmic assurance as an independent pathway to impulsivity. Third, by demonstrating that the dominance of these routes varies across livestream and marketplace contexts, the framework moves beyond linear SOR logic toward a context-contingent, processing-based explanation of digital impulse buying. In contrast, conventional marketplaces foster more reflective, confidence-based processing, resembling the central route. This dual-route adaptation extends prior impulse-buying models (Escobar-Farfán et al., 2025; Kathuria and Bakshi, 2024a) by explicitly integrating AG as both a situational stimulus and a cognitive facilitator.

The remainder of the paper proceeds as follows. We first develop the theoretical framework and hypotheses, then describe the research methodology, followed by the empirical results. We conclude by integrating the findings, discussing theoretical and practical implications, and outlining limitations and directions for future research.

## 2 Literature review and hypothesis development

### 2.1 Algorithmic quality, decision confidence, and impulse buying intentions

Algorithmic quality refers to consumers’ perceptions of the accuracy, relevance, and usefulness of algorithm-generated outputs, such as product recommendations, personalized search results, or predictive cues (Tran et al., 2024; Ram et al., 2024). High-quality algorithmic outputs enhance decision-making efficiency, reduce information overload, and strengthen trust in digital platforms (Nguyen-Viet et al., 2024). When consumers perceive that recommendation systems consistently align with their needs and preferences, they are more likely to develop favorable evaluations and spontaneous purchase tendencies (Jiang et al., 2021). Digital confidence (DC) refers to consumers’ subjective sense of decisional assurance and perceived competence when navigating algorithm-mediated purchasing environments. It reflects an internal cognitive state in which consumers feel sufficiently informed, secure, and capable of proceeding with a transaction. Unlike affective arousal, which triggers spontaneous action, digital confidence operates as a reflective validation mechanism that legitimizes purchase intentions under conditions of uncertainty. From a cognitive-affective processing perspective, AQ enhances DC by reducing uncertainty and making choices appear more accurate and justifiable (Jamil et al., 2025). This sense of psychological assurance can, in turn, facilitate IBI, defined as spontaneous and unplanned purchase inclinations triggered by situational stimuli (Gallin and Portes, 2024; Kathuria and Bakshi, 2024b). Accordingly, we propose the following hypotheses:

*H1a*: Algorithmic quality is positively associated with impulse-buying intentions.

*H1b*: Decision confidence mediates the relationship between algorithmic quality and impulse-buying intentions.

### 2.2 Algorithmic governance, decision confidence, and impulse-buying intentions

Algorithmic governance captures users’ perceptions of transparency, explainability, fairness, and user control embedded in algorithmic systems (Sarkar et al., 2025). While much prior research conceptualizes AG primarily as a risk-mitigating or trust-building mechanism, emerging evidence suggests that transparent governance can also stimulate behavioral engagement (Anoop and Rahman, 2025) and promote consumption by enhancing users’ sense of safety and legitimacy (Lee et al., 2022). In e-commerce settings, visible governance cues such as explanations for recommendations, privacy control dashboards, and accountability notices can strengthen perceived control and DC (Legros et al., 2024). When consumers feel informed and protected, psychological barriers to unplanned purchasing may weaken, increasing the likelihood of impulsive action. From this perspective, AG functions not only as a compliance safeguard but also as a behavioral enabler (Agarwal and Mitra, 2025). Accordingly, algorithmic governance is conceptualized at the consumer-perception level rather than at the institutional or regulatory level. While broader governance frameworks emphasize principles such as fairness, accountability, and contestability, consumers typically infer these principles indirectly through observable interface cues. Accordingly, this study operationalizes algorithmic governance through perceived transparency and perceived privacy protection, which represent the most salient consumer-facing manifestations of governance mechanisms in digital commerce environments. These dimensions capture how governance signals are experienced by users during interaction with algorithmic systems, while other institutional aspects of governance remain outside the empirical scope of the present analysis. Therefore, we hypothesize that:

*H2a*: Algorithmic governance is positively associated with impulse-buying intentions.

*H2b*: Decision confidence mediates the relationship between algorithmic governance and impulse-buying intentions.

### 2.3 Hedonic motivation, decision confidence, and impulse-buying intentions

Hedonic motivation (HM) refers to the enjoyment, pleasure, or fun experienced during interactions with digital technologies (Seng and Hee, 2021). In digital commerce contexts, hedonic motivation energizes affective engagement and has been shown to increase spontaneous purchasing behavior (Mashilo et al., 2025). Enjoyable experiences may also enhance DC by signaling fluency, ease of use, and experiential satisfaction (Aruldoss et al., 2024). However, the strength of this mechanism may vary across shopping contexts. In conventional marketplaces, where consumers evaluate products at their own pace (Manchanda et al., 2024), enjoyment is more likely to reinforce cognitive processing and strengthen confidence before purchase. In contrast, in livestream environments, hedonic experiences may translate more directly into impulsive behavior, bypassing reflective evaluation due to immediacy and social pressure (Platon, 2024). Based on these arguments, we hypothesize:

*H3a*: Hedonic motivation is positively associated with impulse-buying intentions.

*H3b*: Decision confidence mediates the relationship between hedonic motivation and impulse-buying intentions.

### 2.4 Decision confidence and impulse-buying intentions

Decision confidence refers to the degree of assurance and certainty consumers feel regarding their choices (Venciute et al., 2025). Prior research in decision-making and online consumer behavior demonstrates that higher confidence reduces perceived risk and cognitive dissonance, thereby increasing the likelihood of immediate purchasing (Abbasi et al., 2025). In impulse-buying contexts, DC serves as a proximal cognitive driver that translates perceptions of quality, governance, and enjoyment into action (Jamil et al., 2025). Accordingly, we propose:

*H4*: Decision confidence is positively associated with impulse-buying intentions.

### 2.5 Contextual moderation: livestream commerce vs. conventional marketplaces

Consumer decision-making differs substantially between livestream commerce and conventional online marketplaces in terms of temporal dynamics, social interaction, and cognitive load. Livestream environments are characterized by time pressure, social presence, and real-time persuasion (Nikolinakou et al., 2024), whereas marketplaces allow for comparative evaluation and more reflective reasoning (Feng et al., 2024). As a result, the strength and nature of the relationships among AQ, AG, hedonic motivation, DC, and IBI are expected to vary across these contexts (Zhao et al., 2023).

Livestream commerce and conventional marketplaces differ fundamentally in their communicative structure and temporal dynamics. Livestream environments are characterized by real-time interaction, heightened social presence, media richness, and time-limited promotional cues. According to social presence and media richness theories, such environments increase perceptual vividness and emotional engagement while simultaneously imposing cognitive load (Song, 2023). Time pressure and synchronized interaction reduce opportunities for systematic evaluation, thereby favoring heuristic processing.

In contrast, conventional marketplaces provide asynchronous browsing, structured comparison tools, and greater temporal flexibility. These features facilitate deliberative evaluation and reduce reliance on affective immediacy. As a result, consumers in marketplace environments are more likely to engage in reflective processing and seek cognitive assurance before committing to purchase decisions.

Accordingly, the relative dominance of cue-driven versus confidence-mediated pathways is expected to vary across digital commerce contexts (Kizilcec, 2016). Therefore, we expect context-specific differences in the proposed relationships:

*H5a–H5e*: The relationships among algorithmic quality, algorithmic governance, hedonic motivation, decision confidence, and impulse-buying intentions differ significantly between livestream and marketplace contexts.

### 2.6 Dual-process mechanism in algorithm-mediated commerce

Drawing on dual-process theory, consumer decision-making can be understood as operating through two qualitatively distinct cognitive systems: a fast, automatic, affect-driven route (System 1) and a slower, deliberative, reflective route (System 2). While traditional SOR models describe how environmental stimuli influence internal states and behavioral responses, they often do not differentiate between automatic and reflective cognitive processing.

In algorithm-mediated commerce, these two cognitive systems are activated through different technological and psychological mechanisms. The cue-driven route corresponds to heuristic processing, in which consumers respond spontaneously to vivid environmental cues, emotional stimulation, and algorithmic prompts with minimal cognitive elaboration. Algorithmic quality (AQ) enhances sensory vividness and perceptual fluency, while hedonic motivation (HM) heightens affective arousal. Both reduce deliberative scrutiny and facilitate intuitive, immediate action.

In contrast, the confidence-mediated route reflects reflective processing, whereby consumers evaluate perceived governance signals and assess transaction reliability before forming purchase intentions. Algorithmic governance, through transparency and privacy protection cues, enhances perceived predictability and psychological safety. Access to procedural information and system explanations can encourage users to engage in more systematic evaluation of algorithmic outputs, thereby activating reflective processing mechanisms associated with the central route of persuasion (Petty and Cacioppo, 1986). Research on algorithmic transparency further suggests that providing explanations for automated recommendations encourages users to evaluate system outputs more carefully rather than relying solely on heuristic cues (Kizilcec, 2016).

Thus, rather than merely categorizing constructs *post hoc*, the present framework theorizes two distinct cognitive mechanisms through which algorithmic systems influence impulse-buying intention: one affectively driven and heuristic, the other cognitively mediated and confidence-based.

**Figure 1:**
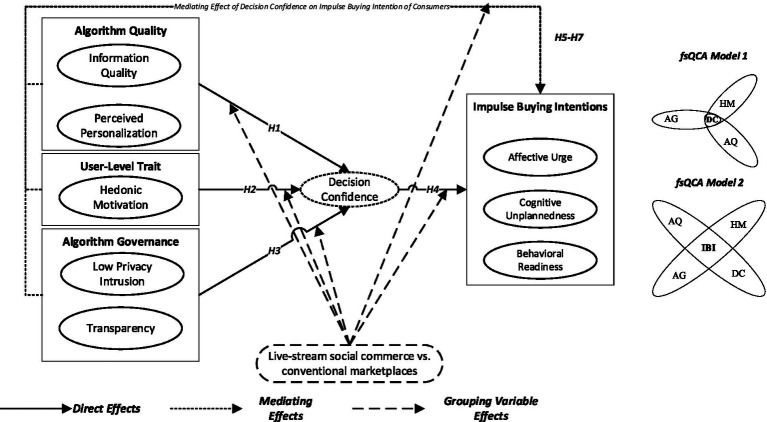
Dual-route model of impulse-buying intentions in algorithm-mediated commerce.

Following Figure 1, the proposed conceptual framework integrates these hypotheses within a dual-route model of impulse buying. Unlike conventional SOR-based impulse-buying models that emphasize affective arousal as the primary internal state, the present framework distinguishes between affective immediacy and cognitive assurance as separate organism-level mechanisms. By integrating dual-process theory, the model specifies not only that stimuli influence internal states, but how different stimuli activate qualitatively distinct modes of cognitive processing. Existing impulse-buying frameworks emphasize heuristic and affective triggers such as website aesthetics or social presence (Imaliya, 2024; Koay and Lim, 2025), but rarely incorporate algorithmic or governance dimensions as systematic stimuli. In contrast, the present model positions AQ and AG as structural antecedents that activate both heuristic (cue-driven) and systematic (confidence-based) processing routes. By synthesizing the impulsive urge perspective (Virvilaitė et al., 2011) with dual-process reasoning, the model illustrates how impulse buying in algorithm-mediated commerce follows distinct cognitive-affective pathways depending on contextual immediacy.

## 3 Methodology

This study employed a quantitative, cross-sectional, between-group design to examine the mediating role of DC in consumers’ IBI across two online shopping contexts: livestream social commerce and conventional online marketplaces. The design allowed for testing both the mediating effect of DC and contextual differences in the relationships among AQ, AG hedonic motivation (HM), and IBI using multi-group analysis (MGA) in SmartPLS 4. In addition, to explore alternative configurational pathways leading to IBI, fsQCA was conducted using two complementary models: (1) AQ, AG, and HM influencing IBI indirectly via DC, and (2) AQ, AG, and HM directly influencing IBI. For fsQCA calibration, we employed the direct calibration method using three qualitative anchors corresponding to full membership, crossover point, and full non-membership. Following established practice for Likert-scale data, the 95th percentile was used as the threshold for full membership, the 50th percentile as the crossover point, and the 5th percentile as the threshold for full non-membership. Calibration values for each construct were computed separately for livestream and marketplace subsamples to maintain contextual comparability.

The target population consisted of Chinese consumers who had engaged in online shopping within the past 6 months. Respondents were recruited through the Wenjuanxing^2^ national online panel over 2 weeks in August 2025. Participants were randomly assigned to either the livestream or marketplace condition to minimize systematic bias. To ensure comparability across contexts, quota balancing was applied ex post, resulting in two equal-sized groups (*n* = 282 per condition). Of the 590 responses initially collected, 26 were excluded due to incomplete responses, straight-lining, failed attention checks, or implausibly short completion times, yielding a final sample of 564 valid observations. All participants were at least 18 years old, provided informed consent before participation, and completed the survey voluntarily.

To establish a shared frame of reference, the questionnaire began with a brief vignette accompanied by visual stimuli corresponding to the assigned shopping context. Each respondent viewed edited screenshots representing the relevant interface. The screenshots were derived from commonly used Chinese platforms (Taobao and JD) and anonymized by blurring brand names, prices, and user identifiers to avoid brand- or seller-specific bias and to protect privacy. Comprehension checks followed the stimulus presentation to ensure participants’ attention before proceeding to the main questionnaire.

All constructs were measured using validated multi-item 7-point Likert-type scales (1 = strongly disagree, 7 = strongly agree) adapted from prior literature and pre-tested for clarity through a bilingual translation and back-translation procedure. All measurement items were adapted from established scales in e-commerce and digital consumption research. To ensure contextual relevance across both livestream and conventional marketplace environments, item wording was carefully modified to reflect real-time interaction features, streamer presence, algorithmic recommendation cues, and platform-specific governance signals. For example, items referencing “platform interface” were adjusted to capture livestream interaction settings where applicable.

Prior to formal data collection, the questionnaire was reviewed by three academic experts in digital marketing and pretested with a pilot sample of online shoppers to ensure clarity, contextual appropriateness, and semantic consistency across both consumption contexts. Minor wording refinements were made based on feedback. These procedures enhance the content validity of the measurement instruments across livestream and marketplace environments. Following Table 1 presents the details of questionnaire development details. The two-stage higher-order construct modeling approach was adopted following Becker et al. (2012) to reduce model complexity and mitigate collinearity. Hedonic motivation was measured using items adapted from UTAUT2 (Venkatesh et al., 2012), with the addition of one context-specific item to capture sustained engagement with the platform; this adaptation was pre-tested and retained for contextual relevance. Also to ensure conceptual alignment between the construct definition and measurement description, the wording used to report item DC4 was clarified to reflect decisional certainty rather than trust attribution.

**Table 1:** Scale development details.

Construct name	Construct definition	Type of variable	No. of items	Scale source (suggested)
Algorithmic Quality	Information Quality, Perceived Personalization	Reflective	4	Adapted from Tran et al. (2024) and Nguyen-Viet et al. (2024)
Algorithmic Governance	Perceived Transparency, Privacy Intrusion	Reflective	4	Adapted from Montecchi et al. (2024) and Alkis and Kose (2022)
Hedonic Motivation	Enjoyment, fun, or pleasure experienced while using the system. Higher = more enjoyment.	Reflective	3	Adapted from UTAUT2 (Venkatesh et al., 2012)
Decision Confidence	Certainty and assurance in one’s choices following system interaction. Higher = more confidence.	Reflective	3	“Decision-making confidence” subscale from Consumer Self-Confidence (Abbasi et al., 2025; Jamil et al., 2025)
Impulse Buying Intentions	Tendency/intention to make spontaneous, unplanned purchases in this context. Higher = stronger impulse buying intention.	Reflective	3–4	Adapted from impulse buying intention/tendency measures (Virvilaitė et al., 2011; Li et al., 2025)

Control variables included age, gender, city tier, monthly online spend, category familiarity, purchase frequency in the focal channel, and the time-of-day/festival week of the purchase. Details are presented below in Table 2.

**Table 2:** Demographic profile of respondents.

		Live stream market		Conventional marketplace	
		Frequency (n)	Percentage (%)	Frequency (n)	Percentage (%)
Gender	Male	118	41.8	123	43.6
	Female	164	58.2	159	56.4
Age (years)	18–25	104	36.9	97	34.4
	26–30	126	44.7	130	46.1
	31–35	52	18.4	55	19.5
Monthly Income (RMB)	2,000–3,000	81	28.7	76	27
	3,100-4,000	119	42.2	121	42.9
	>4,000	82	29.1	85	30.1
Average Purchases per Month	1–2	76	26.9	81	28.7
	3–4	128	45.4	121	42.9
	5 or more	78	27.7	80	28.4
Total		282	100%	282	100%

Several demographic and behavioral variables were included as controls, including age, gender, city tier, monthly online spending, category familiarity, purchase frequency in the focal channel, and the time-of-day or festival period associated with the shopping experience. Procedural remedies for common-method bias included separating predictor, mediator, and outcome blocks with neutral filler items, varying scale anchors, and embedding attention and comprehension checks throughout the survey. Statistical assessments further included marker-variable testing and full collinearity variance inflation factors (VIFs ≤ 3.3). The data were screened for missing values, straight-lining, inconsistent responses, and excessive response speed. Descriptive analyses indicated no problematic outliers, skewness, or kurtosis, and comparisons between early and late respondents revealed no meaningful nonresponse bias.

Hypotheses were tested using PLS-SEM in SmartPLS 4.0, given the exploratory and predictive focus of the model, the presence of higher-order reflective constructs, and evidence of non-normal data distributions. PLS-SEM was preferred over covariance-based SEM because it emphasizes variance explanation and prediction rather than overall model fit and accommodates complex mediation and multi-group structures. Bootstrapping with 5,000 resamples was employed to assess the statistical significance of path coefficients. Measurement invariance across the livestream and marketplace groups was evaluated using the Measurement Invariance of Composite Models (MICOM) procedure before MGA. Contextual differences between groups were then examined using Henseler’s et al. (2015) MGA approach.

To complement the linear modeling results and capture configurational causality, fsQCA was conducted using the fsQCA 4.1 software. All constructs were calibrated into fuzzy sets using the direct method, with percentile-based thresholds defining full membership (95th percentile), crossover points (50th percentile), and full non-membership (5th percentile). Configurations were evaluated based on consistency (≥ 0.80) and coverage to identify sufficient condition combinations leading to high IBI. The use of percentile-based calibration is appropriate for survey data with approximately continuous Likert-type scales and facilitates comparability and internal consistency across conditions (Skillius and Jacobsson, 2024).

## 4 Results

### 4.1 Measurement model

All indicator outer loadings exceeded the recommended threshold of 0.60 in both shopping contexts, supporting indicator reliability. No problematic multicollinearity was detected, as all variance inflation factor (VIF) values were below the recommended cutoff, as reported in Appendix 2. As shown in Table 3, Cronbach’s alpha, composite reliability (CR), rho_A, and average variance extracted (AVE) values for all constructs met or exceeded established thresholds. Specifically, all CR values exceeded 0.70 and all AVEs were above 0.50, indicating satisfactory internal consistency and convergent validity across both livestream and conventional marketplace samples.

**Table 3:** Convergent validity.

Live streaming				
	Cronbach’s alpha	Composite reliability (rho_a)	Composite reliability (rho_c)	Average variance extracted (AVE)
AFURG	0.765	0.784	0.894	0.809
BREAD	0.826	0.839	0.884	0.656
DC	0.874	0.901	0.912	0.722
HM	0.826	0.826	0.896	0.742
IQ	0.776	0.809	0.855	0.597
LPI	0.782	0.779	0.86	0.606
PRSN	0.707	0.718	0.836	0.63
TRP	0.739	0.743	0.852	0.659
UNPLD	0.871	0.877	0.903	0.608

Conventional marketplace				
	Cronbach’s alpha	Composite reliability (rho_a)	Composite reliability (rho_c)	Average variance extracted (AVE)
AFURG	0.799	0.81	0.908	0.832
BREAD	0.803	0.807	0.871	0.629
DC	0.828	0.832	0.885	0.659
HM	0.851	0.858	0.909	0.77
IQ	0.811	0.826	0.875	0.637
LPI	0.752	0.794	0.84	0.572
PRSN	0.78	0.802	0.869	0.689
TRP	0.793	0.836	0.875	0.7
UNPLD	0.858	0.879	0.894	0.585

Discriminant validity was assessed using the Heterotrait–Monotrait (HTMT) ratio criterion. All HTMT values were below the conservative threshold of 0.85 (Henseler et al., 2015), supporting construct distinctiveness. Across both groups, HTMT ratios generally ranged between 0.27 and 0.70. Given that all AVE values exceeded 0.50 in Table 4 and that theoretical distinctions among constructs were clearly specified, all constructs were retained for further analysis.

**Table 4:** Discriminant validity.

Live streaming									
	AFURG	BREAD	DC	HM	IQ	LPI	PRSN	TRP	UNPLD
AFURG									
BREAD	0.72								
DC	0.314	0.613							
HM	0.646	0.574	0.32						
IQ	0.445	0.528	0.286	0.552					
LPI	0.583	0.545	0.442	0.537	0.549				
PRSN	0.626	0.63	0.454	0.494	0.467	0.548			
TRP	0.379	0.267	0.272	0.443	0.609	0.48	0.577		
UNPLD	0.578	0.512	0.411	0.514	0.699	0.856	0.625	0.656	

Conventional marketplace									
	AFURG	BREAD	DC	HM	IQ	LPI	PRSN	TRP	UNPLD
AFURG									
BREAD	0.759								
DC	0.595	0.808							
HM	0.648	0.661	0.55						
IQ	0.431	0.52	0.496	0.547					
LPI	0.291	0.549	0.407	0.307	0.325				
PRSN	0.428	0.556	0.332	0.485	0.47	0.331			
TRP	0.307	0.466	0.405	0.441	0.557	0.384	0.601		
UNPLD	0.541	0.612	0.444	0.517	0.708	0.512	0.664	0.748	

#### 4.1.1 Higher order constructs validation

Several constructs in the model (e.g., Algorithmic Governance and Atmospheric Quality) were specified as reflective–reflective higher-order constructs. This specification is theoretically justified because the lower-order dimensions represent interchangeable manifestations of a broader latent concept and are expected to covary. Changes in the higher-order construct are reflected in changes across its underlying dimensions, consistent with reflective measurement logic.

A reflective-formative specification was not adopted because the dimensions do not constitute independent causal components that collectively form the construct. Rather, they represent correlated expressions of an overarching latent perception. The higher-order constructs were estimated using the repeated indicator approach in SmartPLS, following established guidelines for modeling hierarchical component models in PLS-SEM. The results presented in Table 5 indicate that, in the livestream context, low privacy intrusion contributed more strongly to AG than transparency. In contrast, in the conventional marketplace context, the relative contribution of transparency increased. For IBI, behavioral readiness showed a stronger contribution in the marketplace context, while the three subdimensions remained more evenly balanced in livestream shopping. Overall, these results support the validity of the higher-order construct specifications and justify their use in subsequent structural model estimation and multi-group analysis.

**Table 5:** Higher-order constructs validation results.

Live streaming					
		*β*-value	STDEV	*t*-value	*p*-value
Impulse Buying Intentions (Ca = 0.742, CR = 0.759, AVE = 0.655)	AFURG	0.805	0.041	19.571	***
	BREAD	0.796	0.048	16.69	***
	UNPLD	0.827	0.026	31.893	***
Algorithm Governance (Ca = 0.735, CR = 0.762, AVE = 0.673)	LPI	0.907	0.024	37.267	***
	TRP	0.723	0.096	7.516	***
Algorithm Quality (Ca = 0.816, CR = 0.821, AVE = 0.671)	IQ	0.794	0.057	13.816	***
	PRSN	0.846	0.041	20.806	***

Conventional marketplace					
		*β*-value	STDEV	*t*-value	*p*-value
Impulse Buying Intentions (Ca = 0.767, CR = 0.777, AVE = 0.682)	AFURG	0.803	0.046	17.597	***
	BREAD	0.868	0.027	32.696	***
	UNPLD	0.804	0.045	17.851	***
Algorithm Governance (Ca = 0.847, CR = 0.873, AVE = 0.653)	LPI	0.787	0.059	13.325	***
	TRP	0.829	0.052	15.802	***
Algorithm Quality (Ca = 0.747, CR = 0.752, AVE = 0.688)	IQ	0.852	0.041	20.536	***
	PRSN	0.806	0.066	12.137	***

***Means highly significant effect at *p* < 0.001.

### 4.2 Structural model

The structural model demonstrated adequate explanatory and predictive performance in both shopping contexts. In the livestream model, the predictors accounted for a substantial proportion of variance in IBI. In contrast, in the conventional marketplace model, a larger proportion of variance was explained in DC. Predictive relevance indicators reported in Appendix 4 confirmed acceptable out-of-sample prediction accuracy for both models. Effect size estimates further indicated context-specific patterns, with AQ and AG contributing more strongly to IBI in livestream commerce, and DC playing a more prominent role in the conventional marketplace context (see Appendix 5).

In the livestream group, AG and AQ exhibited significant direct effects on IBI, as well as significant positive effects on DC. The path from DC to IBI was also significant, though smaller in magnitude relative to the direct effects of AG and AQ. Hedonic motivation did not significantly predict DC in this context, but showed a significant direct association with IBI.

In the conventional marketplace group, the path from DC to IBI was strong and significant. Hedonic motivation and AG both showed significant positive effects on DC, whereas the path from AQ to DC was not significant. Direct effects of AG, AQ, and hedonic motivation on IBI remained positive and significant. Table 6 summarizes the results of the direct hypothesis tests for both contexts.

**Table 6:** Hypothesis testing results of direct effects.

Live streaming						
	*β*-value	STDEV	*t*-value	*p*-value	2.50%	97.50%
AG ➔ DC	0.209	0.08	2.61	0.009	0.049	0.363
AG ➔ IBI	0.398	0.075	5.312	0	0.247	0.541
AQ ➔ DC	0.217	0.091	2.374	0.018	0.04	0.397
AQ ➔ IBI	0.3	0.074	4.032	0	0.155	0.453
DC ➔ IBI	0.17	0.046	3.707	0	0.073	0.254
HM ➔ DC	0.06	0.088	0.678	0.498	−0.12	0.225
HM ➔ IBI	0.189	0.059	3.207	0.001	0.08	0.309

Conventional marketplace						
	*β*-value	STDEV	*t*-value	*p*-value	2.50%	97.50%
AG ➔ DC	0.219	0.07	3.119	0.002	0.081	0.358
AG ➔ IBI	0.224	0.074	3.018	0.003	0.078	0.368
AQ ➔ DC	0.144	0.093	1.556	0.12	−0.022	0.345
AQ ➔ IBI	0.289	0.086	3.357	0.001	0.127	0.465
DC ➔ IBI	0.298	0.056	5.32	0	0.175	0.394
HM ➔ DC	0.309	0.084	3.688	0	0.126	0.455
HM ➔ IBI	0.237	0.071	3.332	0.001	0.097	0.374

Mediation effects were assessed using bootstrapping procedures. As shown in Table 7, DC played a limited but statistically significant mediating role in the livestream context for selected paths. In contrast, mediation effects were stronger and more consistent in the conventional marketplace context. These results indicate that the indirect effects via DC varied across shopping environments.

**Table 7:** Hypothesis testing results of indirect (mediation) effects.

Live streaming						
	*β*-value	STDEV	*T* statistics	*p*-values	2.50%	97.50%
AG ➔ DC ➔ IBI	0.036	0.018	1.932	0.053	0.005	0.077
AQ ➔ DC ➔ IBI	0.037	0.017	2.119	0.034	0.006	0.074
HM ➔ DC ➔ IBI	0.01	0.015	0.674	0.501	−0.021	0.041

Conventional marketplace						
	*β*-value	STDEV	*T* statistics	*P*-values	2.50%	97.50%
AG ➔ DC ➔ IBI	0.065	0.026	2.556	0.011	0.019	0.12
AQ ➔ DC ➔ IBI	0.043	0.026	1.623	0.105	−0.006	0.097
HM ➔ DC ➔ IBI	0.092	0.031	2.994	0.003	0.032	0.15

### 4.3 MICOM results

Measurement invariance was evaluated using the Measurement Invariance of Composite Models (MICOM) procedure. Configural invariance (Step 1) was established, as both groups used identical indicators, data treatment procedures, and algorithmic settings. Compositional invariance (Step 2) was also supported, with high composite correlations ranging from 0.979 to 1.000 and non-significant permutation test results for all constructs (e.g., AG = 0.057; AQ = 0.337; DC = 0.413; HM = 0.961; IBI = 0.122), as reported in Appendix 6. For Step 3, equality of means and variances was supported for most constructs, with the exception of hedonic motivation, which exhibited a significant mean difference between groups (*p* = 0.036). Overall, these results satisfy the criteria for partial measurement invariance and support the use of multi-group analysis.

### 4.4 MGA results

Multi-group analysis (MGA) compared structural path coefficients between the livestream and conventional marketplace contexts. As summarized in Table 8, most path differences were not statistically significant, indicating broadly similar structural relationships across contexts. Two exceptions were observed. The path from hedonic motivation to DC differed significantly in the two-tailed test (*p* = 0.043), and the indirect path from hedonic motivation to IBI via DC also showed a significant difference between groups (*p* = 0.012).

**Table 8:** MGA results.

	Difference (LS – CM)	1-tailed (LS vs. CM) *p*-value	2-tailed (LS vs. CM) *p*-value
AG ➔ DC	−0.01	0.535	0.929
AG ➔ IBI	0.174	0.05	0.1
AQ ➔ DC	0.072	0.288	0.576
AQ ➔ IBI	0.011	0.463	0.926
DC ➔ IBI	−0.128	0.96	0.081
HM ➔ DC	−0.249	0.978	0.043
HM ➔ IBI	−0.048	0.699	0.602
AG ➔ DC ➔ IBI	−0.03	0.83	0.34
AQ ➔ DC ➔ IBI	−0.006	0.582	0.837
HM ➔ DC ➔ IBI	−0.082	0.994	0.012

### 4.5 fsQCA results

fsQCA was conducted to identify configurational pathways leading to high digital confidence (DC) and high impulse-buying intention (IBI) across livestream and conventional marketplace contexts. All solution configurations exceeded the recommended consistency threshold of 0.80, with consistency values ranging from 0.87 to 0.94 and raw coverage values between 0.47 and 0.87, indicating strong explanatory adequacy.

For fsQCA calibration, the direct method proposed by Ragin (2008) was employed. Following established practice for Likert-scale data, three qualitative anchors were specified for each construct: full membership, crossover point, and full non-membership. The 95th percentile was used as the threshold for full membership, the 50th percentile as the crossover point, and the 5th percentile as the threshold for full non-membership. Calibration was conducted separately for livestream and marketplace subsamples.

As shown in Table 9, two dominant configurations were sufficient for high digital confidence. In the livestream context (LS–M1), the joint core presence of algorithmic quality (AQ) and algorithmic governance (AG), combined with the absence of hedonic motivation (HM), led to high DC. This configuration suggests that in livestream environments, consumers’ confidence can be established through perceived system reliability and governance mechanisms, even when hedonic stimulation is low.

**Table 9:** fsQCA results.

Conditions	AQ	AG	HM	DC	Outcome	Consistency	Raw cov.	Unique cov.
LS – M1: DC	●	●	⊗		DC	0.893	0.47	–
Editors – M1: DC	●	●	●		DC	0.876	0.562	–
LS – M2: IBI #1	●	●	○	○	IBI	0.943	0.706	0.028
LS – M2: IBI #2	●	○	●	○	IBI	0.932	0.664	0.021
LS – M2: IBI #3	○	●	○	●	IBI	0.929	0.659	0.024
LS – M2: IBI #4	○	○	●	●	IBI	0.922	0.62	0.021
CM – M2: IBI #1	●	●	○	○	IBI	0.933	0.671	0.027
CM – M2: IBI #2	●	○	●	○	IBI	0.94	0.678	0.026
CM – M2: IBI #3	●	○	○	●	IBI	0.926	0.663	0.022
CM – M2: IBI #4	○	●	●	○	IBI	0.933	0.624	0.01
CM – M2: IBI #5	○	●	○	●	IBI	0.928	0.641	0.037

● = core presence; ○ = peripheral presence; ⊗ = absence, blank = do not care.

In the conventional marketplace context (Editors–M1), high DC emerged when AQ, AG, and HM were all present. This indicates that in less immersive environments, digital confidence is jointly shaped by technological assurance and hedonic value, reflecting a more deliberative and evaluative confidence-formation process.

For Model 2, multiple equifinal configurations leading to high IBI were identified in both contexts, demonstrating causal complexity and asymmetry. In the livestream context (LS–M2 #1–#4), high IBI was primarily driven by cue-driven configurations in which AQ and/or HM appeared as core or peripheral conditions, while DC was not consistently required. These patterns indicate that emotionally stimulating cues and immersive technological features can directly trigger impulsive purchasing without reliance on confidence-based deliberation.

In contrast, configurations observed in conventional marketplaces (CM–M2 #1–#5) more frequently incorporated digital confidence and algorithmic governance as core or peripheral conditions. This suggests that in marketplace settings, impulse-buying intentions are more likely to emerge when consumers feel cognitively assured about the transaction process, highlighting the importance of confidence-mediated pathways.

Overall, the fsQCA results complement the PLS-SEM findings by revealing multiple sufficient combinations of cue-driven and confidence-based conditions that lead to high IBI. While PLS-SEM identifies the net effects of individual predictors, fsQCA demonstrates that impulse buying can arise through distinct configurational routes across different digital commerce contexts.

## 5 Discussion

This study advances research on impulse buying in algorithm-mediated commerce by integrating linear and configurational evidence to reveal a dual-route behavioral process. The PLS-SEM results show that AG and AQ are significant positive predictors of IBI in both livestream and conventional marketplace contexts. Hedonic motivation (HM) exerts a stronger direct influence on IBI in marketplaces. At the same time, DC partially mediates the effects of AG and AQ, with mediation more pronounced in conventional marketplaces. Complementing these findings, fsQCA confirms the central role of AQ and AG in shaping both DC and IBI while revealing equifinal pathways not observable in linear models. In livestream commerce, high AQ combined with strong AG is sufficient to yield high DC regardless of HM. In contrast, in marketplaces, the joint presence of AQ, AG, and HM emerges as a dominant configuration. Together, these results demonstrate that SEM and fsQCA are complementary: SEM captures average linear and mediating effects, while fsQCA identifies alternative causal configurations that vary by context.

Building on this integrated evidence, the study moves beyond traditional impulse-buying frameworks by demonstrating that algorithmic systems can trigger impulsive action through both heuristic cues and confidence-driven reasoning. This governance-induced confidence pathway reframes how transparency and algorithmic assurance shape consumer spontaneity. In livestream environments, cue-driven processing dominates, with AQ and AG functioning as salient heuristic cues that stimulate spontaneous purchases under social immediacy and time pressure. In contrast, in conventional marketplaces, a confidence-mediated route emerges in which AG and HM strengthen DC, thereby promoting IBI. The findings are partially consistent with a dual-route interpretation of impulse buying in algorithm-mediated commerce. While the configurational fsQCA analysis suggests that different combinations of technological and motivational conditions can produce high impulse-buying intentions across contexts, the SEM-based multi-group analysis indicates selective rather than comprehensive contextual differentiation. These results therefore provide suggestive rather than definitive evidence of distinct cue-driven and confidence-mediated pathways (Petty, 1986; Wang et al., 2024; Amin, 2025; Zafar et al., 2021), illustrating that impulsive behavior depends on whether consumers process cues peripherally or systematically (Song, 2023; Skillius and Jacobsson, 2024).

The findings extend classic impulse-buying models (Mustikasari et al., 2025; Koay et al., 2021) by explicitly embedding algorithmic features within dual-route logic. Whereas prior research often treats recommendation systems as neutral utilities (Mat Rahim and Goi, 2025; Arrafi and Ghabban, 2021), this study shows that algorithms actively shape cognitive processing modes. High AQ operates as a credibility cue in livestreams and as diagnostic input supporting confidence in marketplaces. At the same time, AG provides transparency and control that can either directly stimulate impulsive action or indirectly enhance DC. Platform algorithms thus function not merely as facilitators of choice but as drivers of processing route selection in digital commerce.

The results also reposition AG from a predominantly protective mechanism to an active behavioral catalyst. Consistent with transparency-trust models (Chen et al., 2025; Li et al., 2023), governance cues enhance confidence and intention in context-specific ways: as heuristic trust signals in livestream commerce and as confidence-building mechanisms in conventional marketplaces. By distinguishing AG from related constructs such as trust and system quality, the study clarifies conceptual boundaries that are often conflated in prior work (Alhelaly et al., 2025; Sarkar et al., 2025). The non-significant effect of algorithmic quality on digital confidence in marketplace settings may reflect consumers’ habituation to mature e-commerce infrastructures. In established online marketplaces, baseline expectations regarding information quality, recommendation relevance, and system reliability are often institutionalized, reducing the incremental influence of algorithmic cues on perceived decision confidence (Gefen et al., 2003; Pavlou and Fygenson, 2006; Kim and Benbasat, 2009). In mature marketplace environments, baseline expectations regarding information quality and personalization may already be institutionalized, reducing their incremental impact on perceived confidence.

Hedonic motivation further reinforces this dual-route distinction. In conventional marketplaces, enjoyment strengthens DC, consistent with utilitarian-hedonic integration perspectives in which pleasurable experiences enhance evaluative assurance (Mashilo et al., 2025; Sumarliah et al., 2022). In livestream environments, affect operates more directly, as excitement translates into immediate purchase urges without reliance on confidence mediation (Jamil et al., 2025). This contrast underscores how platform design shapes the psychological function of enjoyment, with confidence cues playing a more critical role in reflective environments and real-time affective cues dominating in immersive settings.

The stronger cue-driven effects observed in livestream commerce may be explained by heightened temporal immediacy and social presence. Real-time interaction, limited-time promotions, and streamer endorsements increase cognitive load and reduce deliberative processing, thereby amplifying heuristic responses. In contrast, conventional marketplaces allow asynchronous browsing and comparison, which supports reflective evaluation and strengthens the role of confidence-mediated pathways.

The cross-context differences observed in the MGA analysis reflect systematic variation in cognitive processing dominance. In livestream commerce, the stronger direct effects of algorithmic quality and governance on impulse-buying intention suggest that vivid technological cues and real-time prompts can directly activate heuristic processing without requiring cognitive validation. The immediacy and social presence embedded in livestream settings reduce deliberative scrutiny, allowing cue-driven mechanisms to operate more forcefully.

In contrast, in conventional marketplace environments, digital confidence plays a more central mediating role. The asynchronous browsing structure and reduced time pressure encourage reflective evaluation, increasing the importance of cognitive assurance before purchase decisions are made. The non-significant effect of algorithmic quality on digital confidence in marketplace settings may indicate that baseline expectations of system functionality are already institutionalized in mature e-commerce environments, reducing their incremental impact on confidence formation.

The fsQCA findings provide additional insight by confirming equifinality: multiple combinations of AQ, AG, HM, and DC can produce high IBI (Zhang et al., 2022; Xiang et al., 2022). In livestream commerce, AQ and AG alone can generate high confidence even in the absence of enjoyment, reflecting heuristic substitution whereby credible system cues replace affective engagement. In conventional marketplaces, the combination of AQ, AG, and HM produces IBI through confidence-based processing, illustrating complementarity under central-route reasoning. Together, the SEM and fsQCA results reveal context-specific cognitive architectures in which impulse buying arises either from rapid cue assimilation or from confidence-based deliberation, depending on platform dynamics (Verhagen and Van Dolen, 2011). The combined use of PLS-SEM and fsQCA offers complementary insights into impulse-buying mechanisms. PLS-SEM identifies net effects and mediation relationships, whereas fsQCA captures configurational complexity and causal asymmetry. Findings show that digital confidence is not a universally necessary condition for high impulse-buying intention. In livestream commerce, high IBI can emerge primarily from cue-driven conditions without confidence-mediated validation. In contrast, marketplace settings more often rely on digital confidence and governance signals as core elements. This indicates that dominant processing routes are context-contingent and that impulse buying arises from distinct affective–cognitive configurations. Integrating symmetric and asymmetric approaches thus demonstrates that algorithm-mediated impulse buying is multi-causal, non-linear, and context-dependent rather than driven by single predictors.

Finally, the findings contribute to debates on algorithmic transparency and trustworthy artificial intelligence. Governance cues are not merely compliance artifacts; they actively shape consumer behavior by fostering confidence or triggering heuristic trust (Qin et al., 2023; Zhou and Lou, 2024). For regulators, this implies that transparency design should balance clarity with ethical restraint to avoid exploiting consumer spontaneity. For platform designers, features such as explainable recommendation widgets, privacy dashboards, and concise assurance prompts can enhance engagement while supporting responsible AG.

While the present study identifies cue-driven and confidence-mediated pathways to impulse-buying intention across digital commerce contexts, these mechanisms may operate differently under varying situational and individual conditions. First, product involvement is likely to shape the relative dominance of the two routes. For low-involvement products (e.g., inexpensive or hedonic goods), consumers are more likely to rely on heuristic processing, thereby strengthening the cue-driven pathway activated by atmospheric quality and hedonic motivation. In contrast, high-involvement products (e.g., expensive or functional goods) typically trigger more deliberate cognitive evaluation, potentially amplifying the role of digital confidence and algorithmic governance in shaping impulse-buying intentions.

Second, individual differences in trait impulse-buying tendency may moderate the dual-route mechanism. Consumers with high trait impulsivity are more prone to affect-driven and spontaneous decision-making, which may intensify the impact of cue-based stimuli while attenuating the mediating role of digital confidence. Conversely, consumers with lower impulsivity may rely more heavily on cognitive assurance mechanisms before forming purchase intentions.

These potential boundary conditions suggest that the dual-route framework is context-sensitive and contingent on both situational product characteristics and stable consumer traits. Future research may incorporate product involvement and trait impulsivity as moderating variables to further refine the psychological mechanisms underlying digital impulse buying.

In summary, this study develops a dual-route algorithmic framework for impulse buying grounded in dual-process and stimulus-organism-response theories. By reframing AG as an active behavioral driver that operates heuristically in livestream commerce and cognitively in conventional marketplaces, and by demonstrating equifinality and asymmetry through the triangulation of SEM and fsQCA, the study offers a more nuanced understanding of impulsive behavior in digital commerce. Future research should extend these insights using longitudinal or experimental designs and examine additional dimensions of AG, such as fairness and accountability, as higher-order antecedents.

## 6 Implications

### 6.1 Theoretical implications

This study advances impulse-buying theory by conceptualizing algorithm-mediated commerce as a dual-route decision process that integrates cue-driven and confidence-mediated mechanisms. Building on the ELM (Petty, 1986) and the stimulus-organism-response paradigm (Wang et al., 2024), the findings demonstrate that impulsive purchasing can arise from either heuristic cues or reflective confidence, depending on platform context. Livestream environments primarily activate a cue-driven route, in which immediacy, vividness, and algorithmic prompts directly trigger spontaneous action. In contrast, conventional marketplaces follow a confidence-mediated route, whereby AG and hedonic engagement jointly strengthen decision assurance before purchase. This dual-route framing extends existing impulse-buying models that emphasize affective or environmental triggers by identifying algorithmic assurance as a distinct cognitive pathway to impulsivity.

The study also repositions algorithmic governance as an active behavioral catalyst rather than a passive safeguard in digital commerce environments. Consumer-facing governance cues particularly perceived transparency and privacy protection can reduce perceived risk and foster psychological safety during algorithm-mediated shopping interactions. While broader governance principles such as fairness and explainability are frequently discussed in the algorithmic governance literature, these dimensions were not directly captured in the present study and therefore represent important directions for future research. Similarly, AQ functions both as a heuristic credibility signal and as a diagnostic input that enhances DC. Together, these findings clarify how algorithmic systems shape consumer behavior not merely by building trust, but by shifting the dominant mode of information processing between heuristic and deliberative routes.

Methodologically, by integrating PLS-SEM and fsQCA, the study bridges net-effect and configurational perspectives on consumer decision-making. The identification of multiple sufficient causal combinations highlights equifinality and underscores that impulsive behavior emerges from different configurations of technological and psychological conditions across contexts. This hybrid analytical approach advances methodological pluralism in digital commerce research by demonstrating that impulse buying is multi-causal and context-dependent rather than linear or universal.

### 6.2 Managerial implications

For livestream commerce platforms (e.g., Douyin, Kuaishou, Taobao Live), the findings underscore the importance of visible AG and concise system communication in stimulating ethical impulsivity. Transparent governance cues such as disclosure badges, “why recommended” labels, and simple data-control options can foster psychological safety and heighten immediacy without disrupting the flow of interaction. Presenting compact, high-quality information (e.g., brief specifications, review highlights, and authenticity cues) alongside frictionless purchasing mechanisms can help convert attention into spontaneous action. At the same time, platform managers should ensure that urgency tactics, such as countdowns and limited-time offers, remain transparent and compliant with ethical advertising standards to avoid manipulative design practices.

For conventional online marketplaces (e.g., JD, Tmall, and brand-owned platforms), DC emerges as the primary behavioral lever. Confidence-building features, including verified reviews, side-by-side comparisons, clear recommendation explanations, and visible refund or return policies, support deliberative evaluation and strengthen purchase assurance. Governance-oriented user interface elements, such as granular privacy controls and transparent personalization settings, can further enhance conversion when seamlessly embedded in the decision journey. Overall, managers should align interface strategies with the dominant cognitive route of each platform: leveraging real-time heuristic cues in livestream environments and emphasizing reflective reassurance in marketplace settings, while using behavioral analytics to monitor indicators of impulsive versus confidence-driven decision-making.

Importantly, the effectiveness of these strategies depends on the timing and sequencing of interface elements. In livestream environments, heuristic cues such as dynamic viewer counts, limited-time prompts, and animated recommendation tags should be positioned proximal to purchase buttons to capitalize on affective immediacy. In contrast, in marketplace environments, confidence-building elements such as verified reviews, comparison charts, return guarantees, and recommendation explanations should be displayed early in the browsing process to support reflective evaluation before checkout. By aligning the placement and sequence of interface components with the dominant cognitive route of each platform, managers can calibrate impulsivity in a manner that enhances conversion while preserving transparency and user trust.

### 6.3 Policy and regulatory implications

At the regulatory level, the findings indicate that AG cues do more than ensure compliance; they actively shape consumer behavior and therefore function as elements of behavioral architecture. Regulatory frameworks such as China’s Algorithmic Recommendation Regulation and the EU AI Act should consider not only whether transparency and user control mechanisms are present, but also how their timing, visibility, and framing influence consumer impulsivity and consent. Policymakers can promote “trustworthy-by-design” practices by standardizing the placement of disclosures, requiring clear reversibility mechanisms (e.g., undo or cooling-off options), and auditing the behavioral effects of algorithmic explainability features. Such measures help ensure that governance-driven persuasion supports informed autonomy rather than unintended manipulation, thereby maintaining an appropriate balance between innovation and consumer protection.

## 7 Limitations and future work recommendations

This study has several limitations that also suggest directions for future research. First, the use of cross-sectional, self-reported survey data limits causal inference and may introduce common method bias or social desirability bias. Future research could employ experimental or longitudinal designs to better capture the dynamic evolution of impulse-buying mechanisms over time. Moreover, impulse-buying intention was measured through self-report rather than observed behavioral data. Future research could utilize clickstream data, transaction logs, or field experiments to examine whether the identified dual-route mechanisms translate into actual impulsive purchasing behavior.

Second, the sample consists of Chinese consumers interacting with major Chinese platforms (e.g., Taobao Live and JD.com), which may limit generalizability to other cultural contexts or regulatory environments. Differences in privacy norms, governance regulations, and platform architectures may influence the relative importance of cue-driven and confidence-mediated pathways. Third, although we theorized potential boundary conditions such as product involvement and trait impulsivity, these variables were not empirically modeled in the present study. Incorporating situational factors (e.g., promotional intensity, time pressure) and individual differences in future research would further refine the contextual sensitivity of the dual-route framework.

Fourth, while algorithmic governance was conceptually grounded in transparency, fairness, and accountability principles, the operationalization primarily captures consumer-perceived transparency and privacy protection cues. Future studies may develop multidimensional measures that more explicitly differentiate fairness and accountability dimensions. Finally, the present analysis focuses on static configurational patterns. Longitudinal or behavioral trace data could provide deeper insights into how cue-driven and confidence-based mechanisms evolve across repeated platform interactions.

Future research may incorporate algorithmic literacy as a moderating variable within the dual-route framework. Consumers with higher levels of algorithmic literacy may better understand recommendation logic and therefore rely less on surface-level cues, strengthening reflective confidence-based processing. Conversely, lower algorithmic literacy may amplify cue-driven susceptibility in immersive digital environments.

## Data Availability

The raw data supporting the conclusions of this article will be made available by the authors, without undue reservation.

## Footnotes

1
JD.com

2
https://www.wjx.cn/
