# Supplementary material for: A dual-process psychological model of impulse buying in digital commerce: evidence from livestream and marketplace contexts
Source: Front Psychol. 2026 Mar 24;17:1774474. doi: 10.3389/fpsyg.2026.1774474 (PMC13054722; doi:10.3389/fpsyg.2026.1774474)
Supplement: Supplementary file 1 [file Data_Sheet_1.docx]

Appendix 1. Survey Questionnaire

| **Questionnaire** | | | | | | | | |
| --- | --- | --- | --- | --- | --- | --- | --- | --- |
| Dear Participant,  You are invited to participate in a brief academic survey examining consumers’ perceptions of online shopping platforms. You will be shown one shopping interface and asked to answer a few short questions about your impressions and reactions. There are no right or wrong answers; we are only interested in your honest opinions. Your responses will remain strictly confidential and used only for academic research. The survey will take approximately 5-7 minutes.  By proceeding, you confirm that you are at least 18 years old and voluntarily consent to participate in this study.  ☐ I agree to participate. ☐ I do not agree (exit survey). | | | | | | | | |
| **Section 1: Experimental Stimulus** | | | | | | | | |
| You will now see an example of an online shopping interface. Please imagine that you are browsing for a handbag on this platform. Take a moment to view the page carefully before answering the questions that follow. | | | | | | | | |
| 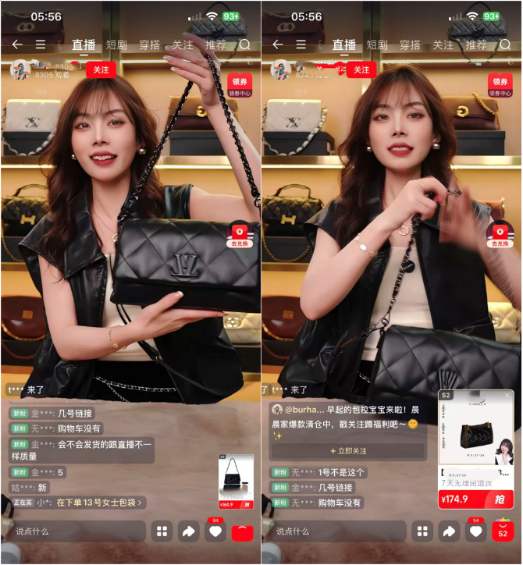  Condition 1: *livestream Shopping Interface* | | | | | | | | |
| 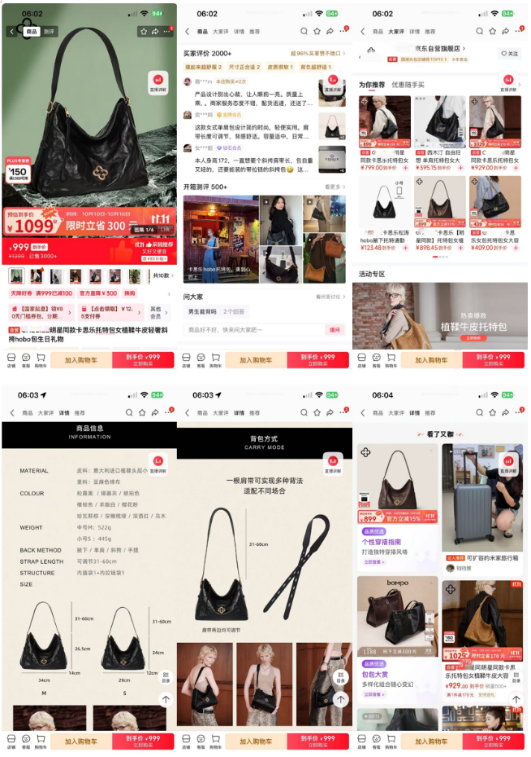  Condition 2: *Conventional Marketplace Interface* | | | | | | | | |
| **Section 2: Questionnaire items for 7-point Likert Scale (1 = Strongly Disagree, 7 = Strongly Agree)** | | | | | | | | |
| **Sr.** | **Item** | **1** | **2** | | **3** | **4** | | **5** |
| **Algorithm Quality (AQ)** | | | | | | | | |
| ***Information Quality*** | | | | | | | | |
| IQ1 | The information provided on the platform was clear and easy to understand. |  |  | |  |  | |  |
| IQ2 | The product information was accurate and reliable. |  |  | |  |  | |  |
| IQ3 | The details on the platform helped me evaluate the product effectively. |  |  | |  |  | |  |
| IQ4 | The information on the platform was comprehensive and covered all the aspects I needed to know. |  |  | |  |  | |  |
| ***Perceived Personalization*** | | | | | | | | |
| PRSN1 | The platform seemed to recommend products according to my preferences. |  |  | |  |  | |  |
| PRSN2 | The information appeared tailored to my interests. |  |  | |  |  | |  |
| PRSN3 | The content shown matched my shopping habits and needs. |  |  | |  |  | |  |
| **Algorithm Governance (AG)** | | | | | | | | |
| ***Low Privacy Intrusion*** | | | | | | | | |
| LPI1 | The platform respected my personal data and privacy. |  |  | |  |  | |  |
| LPI2 | I felt that my personal information would not be misused. |  |  | |  |  | |  |
| LPI3 | The system did not make me feel uncomfortable about privacy. |  |  | |  |  | |  |
| LPI4 | The platform made me feel safe when sharing or using my personal information. |  |  | |  |  | |  |
| ***Transparency*** | | | | | | | | |
| TRP1 | The platform clearly explained why certain products were shown to me. |  |  | |  |  | |  |
| TRP2 | I understood how the system generated product recommendations. |  |  | |  |  | |  |
| TRP3 | The platform appeared transparent in its operations. |  |  | |  |  | |  |
| **Hedonic Motivation (HM)** | | | | | | | | |
| HM1 | I found the shopping experience enjoyable. |  |  | |  |  | |  |
| HM2 | I felt entertained while viewing the product. |  |  | |  |  | |  |
| HM3 | I had fun exploring the shopping interface. |  |  | |  |  | |  |
| HM4 | I would enjoy spending more time using this kind of platform. |  |  | |  |  | |  |
| **Decision Confidence (DC)** | | | | | | | | |
| DC1 | I am confident that I can make a good purchase decision using this platform. |  |  | |  |  | |  |
| DC2 | I feel certain about my product choice on this interface. |  |  | |  |  | |  |
| DC3 | I have no doubts about my ability to choose the right product here. |  |  | |  |  | |  |
| DC4 | I feel sufficiently certain about my choice to proceed with this purchase. |  |  | |  |  | |  |
| **Impulse Buying Intention (IBI)** | | | | | | | | |
| ***Affective Urge*** | | | | | | | | |
| AFRG1 | I suddenly wanted to buy the product after seeing it. |  |  | |  |  | |  |
| AFRG2 | The presentation of the product made me feel an immediate desire to purchase. |  |  | |  |  | |  |
| ***Unplannedness*** | | | | | | | | |
| UNPLD1 | I had not planned to buy this product before viewing the page. |  |  | |  |  | |  |
| UNPLD2 | Buying this product would be a spontaneous decision for me. |  |  | |  |  | |  |
| UNPLD3 | This purchase idea came to my mind only after viewing the platform. |  |  | |  |  | |  |
| UNPLD4 | I did not think about buying this item until I saw it on the platform. |  |  | |  |  | |  |
| UNPLD5 | I decided to buy the product without comparing it to other alternatives. |  |  | |  |  | |  |
| UNPLD6 | My decision to purchase occurred instantly, without much prior thought. |  |  | |  |  | |  |
| ***Behavioral Readiness*** | | | | | | | | |
| BREAD1 | I would probably click “buy now” without much thought. |  |  | |  |  | |  |
| BREAD2 | I felt a strong urge to purchase during the experience. |  |  | |  |  | |  |
| BREAD3 | I was ready to make the purchase immediately after viewing the page. |  |  | |  |  | |  |
| BREAD4 | I would be willing to complete the payment process right away. |  |  | |  |  | |  |
| **Section 3: Demographic Information** | | | | | | | | |
| Please provide some basic information about yourself. | | | | | | | | |
| Gender | ☐ Male | ☐ Female | | ☐ Prefer not to say | | | | |
| Age | ☐ 18-25 | ☐ 26-30 | | ☐ 31-35 | | | ☐ 36 or above | |
| Monthly Income (¥) | ☐ 2000-3000 | ☐ 3100-4000 | | ☐ Above 4000 | | | | |
| Average Purchases per Month (on this type of platform) | ☐ 1-2 | ☐ 3-5 | | ☐ 6 or more | | | | |
| Thank you for participating in this study. Your responses have been recorded anonymously and will contribute to ongoing academic research on digital consumer behavior. If you have any questions or wish to receive a summary of results, please get in touch with the researcher. | | | | | | | | |

Appendix 2. Measurement Model Reliability Results

|  |  | **Live Stream** | | **Conventional Marketplace** | |
| --- | --- | --- | --- | --- | --- |
| **Constructs** | **Items** | **OL** | **VIF** | **OL** | **VIF** |
| Affective Urge | AFURG1 | 0.879 | 1.624 | 0.925 | 1.797 |
|  | AFURG2 | 0.919 | 1.624 | 0.9 | 1.797 |
| Behavioral Readiness | BREAD1 | 0.821 | 2.239 | 0.821 | 1.76 |
|  | BREAD2 | 0.803 | 1.71 | 0.73 | 1.466 |
|  | BREAD3 | 0.865 | 2.244 | 0.805 | 1.715 |
|  | BREAD4 | 0.745 | 1.805 | 0.813 | 1.822 |
| Decision Confidence | DC1 | 0.872 | 3.193 | 0.795 | 1.77 |
|  | DC2 | 0.896 | 3.244 | 0.829 | 1.995 |
|  | DC3 | 0.862 | 4.437 | 0.81 | 2.31 |
|  | DC4 | 0.763 | 3.565 | 0.812 | 2.131 |
| Hedonic Motivation | HM1 | 0.893 | 2.258 | 0.886 | 2.167 |
|  | HM2 | 0.838 | 1.712 | 0.891 | 2.117 |
|  | HM3 | 0.852 | 1.909 | 0.856 | 1.984 |
| Information Quality | IQ1 | 0.688 | 1.487 | 0.775 | 1.702 |
|  | IQ2 | 0.846 | 1.789 | 0.742 | 1.656 |
|  | IQ3 | 0.805 | 1.951 | 0.841 | 2.007 |
|  | IQ4 | 0.743 | 1.807 | 0.83 | 1.85 |
| Low Privacy Intrusion | LPI1 | 0.695 | 1.25 | 0.64 | 1.236 |
|  | LPI2 | 0.811 | 2.073 | 0.889 | 2.133 |
|  | LPI3 | 0.793 | 1.658 | 0.676 | 1.46 |
|  | LPI4 | 0.808 | 2.046 | 0.796 | 1.934 |
| Personalization | PRSN1 | 0.815 | 1.595 | 0.86 | 2.095 |
|  | PRSN2 | 0.731 | 1.26 | 0.826 | 1.363 |
|  | PRSN3 | 0.831 | 1.471 | 0.802 | 1.937 |
| Transparency | TRP1 | 0.766 | 1.353 | 0.862 | 1.5 |
|  | TRP2 | 0.798 | 1.539 | 0.836 | 1.967 |
|  | TRP3 | 0.868 | 1.781 | 0.812 | 1.785 |
| Unplannedness | UNPLD1 | 0.746 | 1.824 | 0.737 | 1.781 |
|  | UNPLD2 | 0.756 | 1.934 | 0.795 | 2.173 |
|  | UNPLD3 | 0.821 | 2.186 | 0.848 | 2.428 |
|  | UNPLD4 | 0.807 | 2.116 | 0.806 | 2.091 |
|  | UNPLD5 | 0.796 | 1.837 | 0.735 | 1.704 |
|  | UNPLD6 | 0.748 | 1.703 | 0.655 | 1.484 |

Appendix 3. Descriptives of constructs

|  | **Live Stream** | | **Conventional Marketplace** | |
| --- | --- | --- | --- | --- |
| **Constructs** | **Mean** | **Standard Deviation** | **Mean** | **Standard Deviation** |
| Affective Urge | 4.272 | 0.715 | 4.184 | 0.885 |
| Behavioral Readiness | 4.105 | 0.664 | 4.146 | 0.681 |
| Decision Confidence | 4.971 | 0.979 | 4.074 | 0.916 |
| Hedonic Motivation | 4.247 | 0.597 | 4.117 | 0.743 |
| Information Quality | 4.328 | 0.561 | 4.207 | 0.653 |
| Low Privacy Intrusion | 4.013 | 0.769 | 4.017 | 0.733 |
| Personalization | 4.261 | 0.650 | 4.214 | 0.781 |
| Transparency | 4.317 | 0.589 | 4.182 | 0.739 |
| Unplannedness | 4.212 | 0.675 | 4.140 | 0.745 |

Appendix 4. Model Predictive Power

| **Live Streaming** | | | | | |
| --- | --- | --- | --- | --- | --- |
|  | **R-square** | **R-square adjusted** | **Q²predict** | **RMSE** | **MAE** |
| DC | 0.176 | 0.167 | 0.139 | 0.934 | 0.747 |
| IBI | 0.696 | 0.691 | 0.65 | 0.603 | 0.392 |
| **Conventional Marketplace** | | | | | |
|  | **R-square** | **R-square adjusted** | **Q²predict** | **RMSE** | **MAE** |
| DC | 0.298 | 0.287 | 0.256 | 0.871 | 0.709 |
| IBI | 0.657 | 0.65 | 0.551 | 0.688 | 0.437 |

Appendix 5. Effect Sizes

|  | **Live streaming** | **Conventional Marketplace** |
| --- | --- | --- |
|  | **F-square** | **F-square** |
| AG 🡪 DC | 0.031 | 0.047 |
| AG 🡪 IBI | 0.291 | 0.096 |
| AQ 🡪 DC | 0.032 | 0.018 |
| AQ 🡪 IBI | 0.162 | 0.141 |
| DC 🡪 IBI | 0.078 | 0.182 |
| HM 🡪 DC | 0.003 | 0.097 |
| HM 🡪 IBI | 0.082 | 0.107 |

Appendix 6. MICOM Results

| **Step 2** | | | | | | |
| --- | --- | --- | --- | --- | --- | --- |
|  | **Original correlation** | **Correlation permutation mean** | **5.00%** | **Permutation p value** |  |  |
| AG | 0.979 | 0.994 | 0.98 | 0.057 |  |  |
| AQ | 0.996 | 0.996 | 0.987 | 0.337 |  |  |
| DC | 0.998 | 0.998 | 0.992 | 0.413 |  |  |
| HM | 1 | 0.999 | 0.998 | 0.961 |  |  |
| IBI | 0.998 | 0.999 | 0.997 | 0.122 |  |  |
| **Step 3a** | | | | | | |
|  | **Original difference** | **Permutation mean difference** | **2.50%** | **97.50%** | **Permutation p value** | |
| AG | 0.108 | -0.002 | -0.182 | 0.178 | 0.233 |  |
| AQ | 0.144 | -0.001 | -0.17 | 0.172 | 0.12 |  |
| DC | -0.083 | -0.003 | -0.187 | 0.188 | 0.397 |  |
| HM | 0.196 | -0.003 | -0.186 | 0.185 | 0.036 |  |
| IBI | 0.062 | 0 | -0.17 | 0.181 | 0.51 |  |
| **Step 3b** | | | | | | |
|  | **Original difference** | **Permutation mean difference** | **2.50%** | **97.50%** | **Permutation p value** | |
| AG | -0.066 | 0.004 | -0.44 | 0.459 | 0.762 |  |
| AQ | -0.361 | 0.01 | -0.528 | 0.6 | 0.238 |  |
| DC | -0.079 | 0.004 | -0.255 | 0.275 | 0.561 |  |
| HM | -0.441 | 0.024 | -0.464 | 0.561 | 0.089 |  |
| IBI | -0.235 | 0.006 | -0.531 | 0.524 | 0.386 |  |

Appendix 7. fsQCA Configurations

| **Configuration (Solution term)** | **Raw Cov.** | **Unique Cov.** | **Consistency** |
| --- | --- | --- | --- |
| **LS: M1-DC:** AQ● AG● ~HM⊗ | 0.47 | - | 0.893 |
| **CM: M1-DC:** AQ● AG● HM● | 0.562 | - | 0.876 |
| **LS: M2-IBI:** AQ● AG● + AQ● HM● + AG● DC● + HM● DC● | 0.872 | 0.021-0.028 | 0.874 |
| **CM: M2-IBI:** AQ● AG● + AQ● HM● + AQ● DC● + AG● HM● + AG● DC● | 0.860 | 0.010-0.037 | 0.865 |
